# A comparative study of improvements Pre-filter methods bring on feature selection using microarray data

**DOI:** 10.1186/2047-2501-2-7

**Published:** 2014-10-16

**Authors:** Yingying Wang, Xiaomao Fan, Yunpeng Cai

**Affiliations:** Research Center for Biomedical Information, Shenzhen Institutes of Advanced Technologies, Chinese Academy of Sciences, Shenzhen, China

**Keywords:** Comparative study, Feature selection, Microarray

## Abstract

**Background:**

Feature selection techniques have become an apparent need in biomarker discoveries with the development of microarray. However, the high dimensional nature of microarray made feature selection become time-consuming. To overcome such difficulties, filter data according to the background knowledge before applying feature selection techniques has become a hot topic in microarray analysis. Different methods may affect final results greatly, thus it is important to evaluate these pre-filter methods in a system way.

**Methods:**

In this paper, we compared the performance of statistical-based, biological-based pre-filter methods and the combination of them on microRNA-mRNA parallel expression profiles using L1 logistic regression as feature selection techniques. Four types of data were built for both microRNA and mRNA expression profiles.

**Results:**

Results showed that pre-filter methods could reduce the number of features greatly for both mRNA and microRNA expression datasets. The features selected after pre-filter procedures were shown to be significant in biological levels such as biology process and microRNA functions. Analyses of classification performance based on precision showed the pre-filter methods were necessary when the number of raw features was much bigger than that of samples. All the computing time was greatly shortened after pre-filter procedures.

**Conclusions:**

With similar or better classification improvements, less but biological significant features, pre-filter-based feature selection should be taken into consideration if researchers need fast results when facing complex computing problems in bioinformatics.

**Electronic supplementary material:**

The online version of this article (doi:10.1186/2047-2501-2-7) contains supplementary material, which is available to authorized users.

## Background

During the last decade, feature selection techniques have become an apparent need in many biological and medical analyses fields [1, 2]. With the development of experimental molecular biology, scientists could detect the expression of molecular on ‘omics’ scale. Microarray is one of the most widely used high-throughput techniques genome-wide. Probes are often designed based on messenger RNA (mRNA) transcripts and/or microRNAs (a class of small, non-coding RNAs that play important regulation roles by targeting hundreds or even thousands of target genes) thus make the analyses of mRNA and/or microRNAs expression profiles become one of the hot topics in many fields such as biomarker discovery [3–5], disease relationships [6–8], molecular ranking [9–11], and biological network construction [12, 13], etc. Biomarkers often refer to molecular such as genes, proteins, microRNAs, etc. that could represent the characteristics which is objectively measured and evaluated as an indicator of normal biological processes, pathogenic processes, or pharmacologic responses to a therapeutic intervention [14]. The biomarkers identified from these datasets are often the most discriminating features for classification between different biological conditions or disease stages [15–17]. Such procedures are considered as feature selection in machine learning related fields. The popular feature selection methods can be broadly categorized into the 3 types: filter methods [18, 19], wrapper methods [20, 21], and embedded methods [22]. These methods could help improving disease classification and diagnosis at molecular levels [23–25].

However, the expression data sets generated by microarray technology are often composed of a large number of molecular as potential features compared with a limited number of samples. The expression profile data are often described as a matrix in bioinformatics with rows representing features and columns representing samples. Due to the limitation of many factors such as the cost and ethics of acquiring large number of samples from patients, it is difficult to make the data suitable for existing feature selection algorithms. Feature selection techniques are used in microarray data analyses through selecting a small subset of molecular by removing relatively redundant, noisy, and irrelevant part of the data. However, the high dimensional nature of microarray made feature selection become time-consuming processes.

To overcome such difficulties, filter some features according to the characteristics of data before applying feature selection techniques which we named ‘pre-filter’ procedures in this paper is a good choice. Considering the characters of bioinformatics, researches started to reduce features based on background knowledge in the fields of biology, medicine, and statistics, etc. Thus, many pre-filter methods had been proposed based on statistical or biological considerations as follows: (1) statistical-based pre-filter methods: using statistical methods to find out the differential expressed molecular among different conditions. These procedures are usually simple and fast. Take differential expression molecular identifications as an example, researches used statistical methods to find molecular with expression values fluctuated among different conditions. It is often the first step of microarray analyses and is also one of the most commonly used pre-filter methods. In such kind of procedure, statistical test such as t-test and ANOVA are usually chosen (according to the number of different conditions) due to their stability and easy operability. However, these procedures may often identify features that are isolation from the others. (2) biological-based pre-filter methods: using enrichment analysis based on biological function and/or pathway information to find out potential disease-related molecular. One of the most important goals of microarray analyses is finding the biomarkers with significant biological meanings. Gene Ontology (GO) [26] is composed of three domains BP (Biological Process), MF (Molecular Function), and CC (Cellular Component), all of the which are widely used in functional related analyses. Besides this, the interactions among molecular also contribute greatly to the biological phenomenon. In bioinformatics, these relationships are often exhibited in the form of networks such as biological pathways which could reflect the structure of some biological processes in a systematic way. Several researches of microarray feature selection have added metabolic and/or molecular interaction pathways into their methods such as BPFS (Biological Pathway based Feature Selection algorithm), etc. [27–30]. (3) Combination of statistical and biological-based pre-filter methods. Considering the advantages of the two pre-filter methods mentioned above, some algorithms were designed based on both of them [31].

However, it is unclear to us that how much improvement these pre-filter methods could bring on the feature selection results. In this paper, we compared the performance of these pre-filter methods on 4 microRNA and 10 mRNA microarray datasets. L1 logistic regression was used as the representation of feature selection methods to perform the analyses after pre-filter procedures. All the samples’ class labels were known and used to evaluate the results by using 5-fold cross validation. Our results showed that both of the two kinds of filter methods could increase classification precision slightly while the combination of them could increase the AUC (Area Under Curve) of ROC (Receiver Operating Characteristic) curve slightly. The features were found to be significant on biological levels. All the computing times were shortened greatly.

## Methods

### Microarray datasets

microRNA and mRNA expression profiles of human hypertrophic cardiomyopathy (HCM) were downloaded from NCBI GEO [32] (GSE36961and GSE36946). Samples with both microRNA and mRNA taken from a same person were collected from 106 HCM patients and 20 healthy donors. The raw microRNA microarray data contained 1145 probes which could be mapped to 819 mature microRNAs. The raw mRNA microarray data contained 37846 probes which could be mapped to 18756 Ensembl genes.

### Construction of mRNA datasets

**Figure 1:**
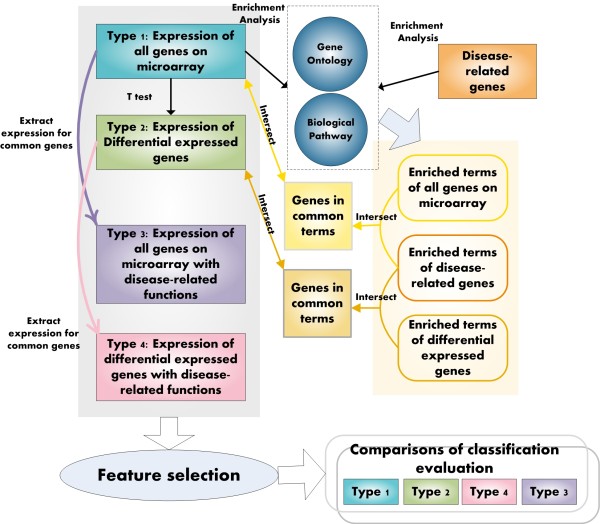
**Framework of mRNA analysis in this study.** Tangles filled with different colors represented the 4 types of mRNA datasets (dark green represents Type1, light green represents Type2, purple represents Type3, and pink represents Type4) while lines with different colors represent 3 kinds of analyses (light and dark orange represent intersect, purple and pink represent extracting expression for common genes, and black represents enrichment analysis).

4 types of mRNA datasets were built as follows (See Figure 1 for details)

-  Type 1: Expression of all genes on microarray. This dataset was built by mapping all the 37846 probes on microarray to 18756 Ensembl genes. All the corresponding expressions in all the samples of these genes were extracted and constructed as type1 mRNA dataset.
-  Type 2: Expression of differential expressed genes. Differential expression genes (DEG) were selected based on t-test, with threshold 0.05. Genes with *p-value* not over 0.05 were chosen as DEGs and their expressions were extracted from raw data to build the type2 mRNA dataset.
-  Type 3: Expression of all genes on microarray with disease related functions. 372 validated HCM related genes were collected from GeneCards [33] and GAD (Genetic Association Database) [34]. The terms of 3 domains of GO were included in this study: 5140 BP terms, 2782 MF terms, and 851 CC terms. 2999 biological pathways were downloaded from several online databases including BioCarta [35], KEGG [36], Pathway Interaction Database [37], and Reactome [38]. The 372 HCM related genes and all genes on microarray were annotated to GO and biological pathways by enrichment analysis using hyper-geometric test with threshold 0.05, separately. GO terms and biological pathways with p-value not above 0.05 were chosen as enriched terms and pathways (See the following part of ‘Method’ for the detail procedure of enrichment analysis). Genes annotated to the same GO terms or biological pathways of validated HCM related genes were picked out and their expressions were extracted to construct the type3 mRNA datasets. 4 datasets were built for such type and named as type3-BP, type3-MF, type3-CC, and type3-Pathway separately.
-  Type 4: Expression of differential expressed genes with disease related functions. Similar to the construction processes of type3, these 4 datasets were built by picking out DEGs annotated to the same GO terms (including BP, MF, and CC terms) or biological pathways of validated HCM related genes. These 4 datasets were named as type4-BP, type4-MF, type4-CC, and type4-Pathway, correspondingly.

### Construction of microRNA dataset

4 types of microRNA datasets were built as follows (See Additional file 1: Figure S1 for details):

-  Type 1: Expression of all microRNAs on microarray. This dataset was built by mapping all the 1145 probes on microarray to 819 mature human microRNAs. Their corresponding expression values in all the samples were extracted to construct the type1 microRNA dataset.
-  Type 2: Expression of differential expressed microRNAs. Differential expression microRNAs (DEM) were selected based on t-test, with threshold 0.05. The expression values of the microRNAs with p-value not over 0.05 were extracted from all the samples to build the type2 microRNA dataset.
-  Type 3: Expression of all microRNAs on microarray with validated disease related genes as targets. 19550 validated microRNA-mRNA relationships were downloaded from mirTarBase [39]. MicroRNAs that regulate at least one validated HCM gene were selected as potential features and their expressions were extracted from all the samples to build this type3 microRNA dataset.
-  Type 4: Expression of differential expressed microRNAs with validated disease related genes as targets. Similar to the construction processes of type3, the expression values in all the samples of DEMs with at least one validated HCM related gene were chosen to build the type4 microRNA dataset.

### Enrichment analysis

Enrichment analysis was used to find functional interpretation for a list of genes chosen by some criteria such as differential expressed in this study. Hyper-geometric test was adopted to perform the analysis with null hypothesis that a functional term (such as GO or biological pathway in this study) was irrelevant to the gene lists. For each functional term and gene list, the *p* value was calculated as follows:


Of which, *a* was the number of genes annotated to a certain functional term, *b* was the total number of genes, d was the number of genes in the list, and *n* was the number of genes in the list annotated to this functional term. All the functional terms with *p* value not above 0.05 were chosen as enriched terms.

### Feature selection

We used *L1* logistic regression to perform the feature selection procedures due to its ability to dispose the high dimensional data [40]. The model describes were as follows:

Let  denoted the dataset, where *x*^n^ ∈ *R*^*N*^ was the *n*-th feature and y_*n*_ ∈ *R*^*N*^ was the label of the *n*-th sample. We used (*w,b*) as the coefficients and intercept of *L*1 logistic regression. The *L1* logistic regression model was listed as follows:


where *L*(.) was the loss function and *λ* was a regularization parameter which had the ability to dispose high dimensional data.

### Evaluation of classification results

5-fold cross validation was used to analyze the classification results of *L1* logistic regression on all the 14 datasets as illustrated above (10 mRNA datasets and 4 microRNA datasets). Three measures including AUC value, precision, and computing time were computed and compared for these test datasets.

A receiver operating characteristic (ROC) was a graphical illustrates the performance of a classifier with the discrimination threshold varied. The area under the curve (AUC) was equal to the probability that a classifier would rank a randomly chosen positive instance higher than a randomly chosen negative one [41]. A bigger AUC meant a ROC close to the left-top of the plot.

Let TP and FP stand for true positives and false positives, the precision was calculated as:


## Results and discussion

### Effects of pre-filter methods on reducing feature dimension

**Figure 2:**
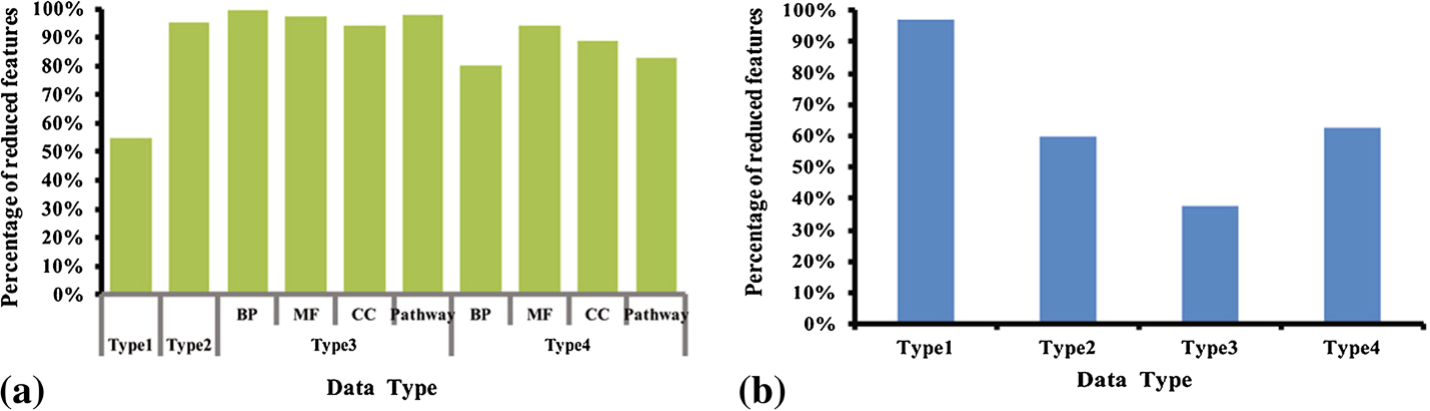
**The percentages of selected features in the raw data after pre-filter procedures. (a)** Results of 10 mRNA datasets **(b)** Results of 4 microRNA datasets. The x-axis represents the names of data type and y-axis represents the percentages that selected features taken in the whole raw datasets, respectively.

4 types including 10 datasets were built for the mRNA expression profiles (See ‘Methods’ for details). The detailed information for the number of raw variables in each set could be found in Table 1. Statistical-based pre-filter methods (Type2) reduced 80.78% features from the raw dataset (Type1) (See Table 1 for details). Biological-based pre-filter methods (Type3) reduced part of raw features as follows: GO-BP 43.79%, GO-MF 40.58%, GO-CC 43.07%, and pathway 27.47%. The combination of the two pre-filter methods reduced features greatly: GO-BP 88.72%, GO-MF 88.10%, GO-CC 88.62%, and pathway 85.03%. After feature selection procedure, the selected features in all the datasets were only a small percentage (See Table 1 and Figure 2(a) for details).

**Table 1:** **Datasets built for mRNA expression profile**

	Type 1	Type 2	Type 3				Type 4			
			***BP***	***MF***	***CC***	***Pathway***	***BP***	***MF***	***CC***	***Pathway***
**Raw variables**	18756	3604	10542	11149	10678	13603	2116	2232	2135	2808
**Selected features**	8465	168	60	270	628	248	416	131	239	482

For the 4 microRNA datasets, both the statistical-based and biological-based pre-filter methods could reduce the number of features greatly while the combination of them could extract 8 microRNAs from all the 819 mature microRNAs (See Table 2 and Figure 2(b) for details). Type 2, type 3, and type 4 datasets reduced 86.08%, 95.48%, and 99.02% of raw features, respectively.

**Table 2:** **Datasets built for microRNA expression profile**

	Type 1	Type 2	Type 3	Type 4
**Raw variables**	819	114	37	8
**Selected features**	23	46	23	3
**AUC**	0.5990566	0.5698113	0.5216981	0.4358491
**Precision**	0.6031746	0.6190476	0.4761905	0.3968254
**Computing time(second)**	61.424	26.986	26.972	12.911

### Effects of pre-filter methods’ influence on biological level

**Figure 3:**
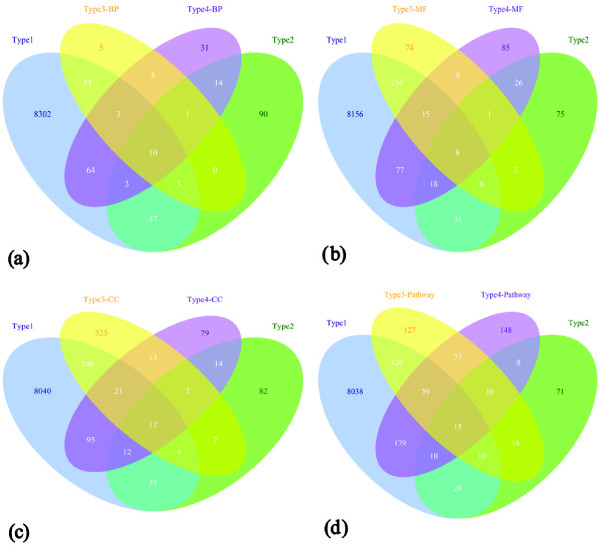
**Overlaps among the selected features of the 10 mRNA datasets on 4 levels.** The numbers in the figures stand for the numbers of common features between the corresponding datasets. **(a)** Overlaps on GO-BP level; **(b)** Overlaps on GO-MF level; **(c)** Overlaps on GO-CC level; **(d)** Overlaps on GO-Pathway level.

There were overlaps among the selected features of the 10 mRNA datasets (As shown in Figure 3(a-d)). For datasets constructed based on GO-BP, the numbers of shared genes were big. Only 8.3% and 7.45% genes in type3-BP and type 4-BP were covered by one datasets of type 1, type 2, typ 3-BP, and type 4-BP. It was interesting that type 3-BP dataset kept only 60 genes as selected genes; however, these genes were enriched in 67.19% of HCM related genes’ enriched GO BP terms. Of these terms, we could see the important biological processes related to HCM such as adult heart development (GO: 0007512), cardiac muscle tissue development (GO: 0048738), muscle system process (GO: 0003012), vasculature development (GO: 0001944), and vasculogenesis (GO: 001570), etc. were covered in this dataset. 55 of these 60 genes were covered by type 1, type 2, and type 4-BP datasets as shown in Figure 3(a). Compared with GO-BP, datasets constructed based on GO-MF showed different results especially type 4-MF, of which only 35.11% genes were in the overlaps among 4 datasets type 1, type 2, typ 3-MF, and type 4-MF (See Figure 3(b) for details). Nearly half of the selected genes (48.57% and 48.79%, respectively) appeared at least twice in type 3-CC and type 3-Pathway (See Figure 3(c-d)). In type 4-CC and type 4-Pathway, over 66% of the selected genes (66.95% and 69.29%, respectively) appeared at least twice (See Figure 3(c-d)).

Compared with mRNA datasets, the numbers of selected microRNAs as features in different datasets were small. The overlaps among them could be seen from Additional file 1: Figure S1. There were 7 microRNAs appearing at least twice in the four datasets as follows: hsa-miR-10a*, hsa-miR-193b*, hsa-miR-302a, hsa-miR-375, hsa-miR-346, hsa-miR-542-3p, and hsa-miR-34c-5p. All the 7 microRNAs were found to be related to HCM to some degree. The expression values of has-miR-10a changed during the latter stage of cardiac hypertrophy [42] and may play an important role in cardiovascular disease [43, 44] which indicated that hsa-miR-10a* may also be a related molecular to HCM. Hsa-miR-193b had been shown to dys-regulated in five or more types of muscular disorders [45] which may also involved in the generation of HCM. Hsa-miR-302a was a tumor-suppressor microRNA, which may be activated by some inhibitors [46]. MiR-375 was one of the most highly expressed microRNAs in 4 key time-points of the fetal mouse heart development [47] indicating it may also play a role in other heart related processes. The over-expression of miR-346 activated the Wnt/β-catenin pathway [48] and this pathway was critical for maladaptive cardiac hypertrophy [49]. Thus hsa-miR-346 may involve in HCM related procedures through regulating the Wnt/β-catenin pathway. Research showed that miR-542-3p was an important positive regulator of p53 [50] and the expression of miR-34c was robustly induced in a p53-dependent manner [51]. The expression of p53 was proved to be increased in HCM patients [52] thus indicating that hsa-miR-542-3p and hsa-miR-34c may participate indirectly in HCM related biological processes through p53.

### Effects of pre-filter methods on classification performance

**Figure 4:**
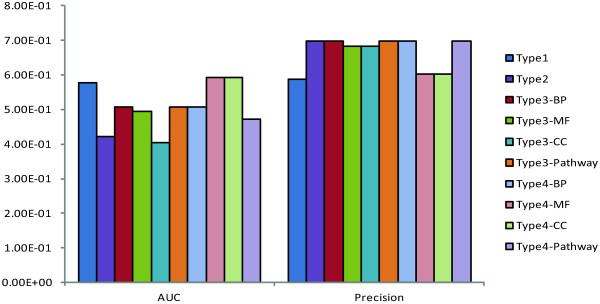
**Comparison of AUC and classification precision of 4 types of mRNA data.** The bars in the graph are clustered in two groups: the left group marked as ‘AUC’ on the x-axes means the height of the bars stand for the AUC values of each dataset while the right group marked as ‘Precision’ on the x-axes means the height of the bars stand for the precision values of each dataset. The 10 datasets are represented with 10 different colors as shown in the figure legend.

Our results showed that for mRNA expression profiles, the pre-filter methods could increase the classification precision (See Methods for the calculation of precision). These indicated that pre-filter methods may improve the performance of feature selection techniques on samples’ positive prediction levels. From Figure 4, we could see that the precision of raw dataset (Type 1) was 0.587 while precisions of other 9 datasets were at least 0.60 (type 4-MF and type 4-CC). However, only combined pre-filter methods increased the AUC values slightly (from 0.5764151 of type 1 to 0. 5933962 of type 4-MF and type 4-CC).

The performance of pre-filter methods on microRNA expression profile did not show similar results with mRNA (See Table 2 for details). All the pre-filter methods did not show an improvement on AUC values which may partly due to the small number of features Type 2-4 contain. Only Type 2 could improve the precision slightly (from 0.6031746 to 0.6190476). In type 1, the number of features was only 6.5 fold bigger of the number of samples. These may indicate us that the pre-filter methods may more suitable to high dimensional data with the number of samples much bigger than features. However, though the evaluations from machine learning level seemed that the pre-filter procedures may not necessary for such small datasets, a good choice was to combine these results generated by different pre-filter methods since the 7 microRNAs (appeared at least twice in the 4 datasets) showed significant biological meanings.

### Effects of pre-filter methods on computing time

**Figure 5:**
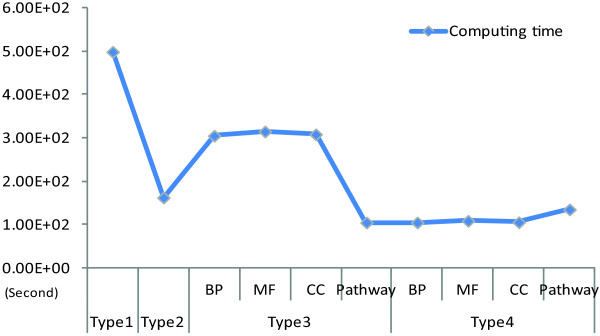
**Comparison of computing time for 4 types of mRNA datasets.** The points stand for the computing time of all the 4 types of mRNA data which including 10 datasets.

All the computing time were shortened after the pre-filter methods used in this paper (See Table 2 and Figure 5 for details). For mRNA datasets, the feature selection procedure spent 498.53 seconds on Type 1, 162.568 seconds on Type 2, 258.28 seconds on Type 3 (average value), and 113.64 seconds on Type 4 (average value). These indicated us that a dramatically advantage of applying pre-filter methods before feature selection was the shortening of computing time.

With similar or better classification improvements, less but with biological significance features, pre-filter-based feature selection should be taken into consideration if researchers needed fast results when facing complex computing problems in bioinformatics.

## Conclusions

Feature selection techniques were often time-consuming when applied on microarray datasets without filters. Our results showed that pre-filter methods could reduce the computing time of the procedure while keeping or improving precision compared with the results of feature selection based on raw datasets.

## Authors’ information

YYW: Ph.D., assistant professor.

XMF: Ph.D., candidate, engineer.

YPC: Ph.D., associate professor.

## Electronic supplementary material

Additional file 1: **Framework of microRNA analysis in this study.** (JPEG 2 MB)

## Abbreviations

ANOVA: ANalysis of vAriance
AUC: Area under curve
BP: Biological Process
CC: Cellular Component
DEG: Differential Expression Gene
DEM: Differential Expression microRNA
FP: False Positive
GO: Gene Ontology
HCM: Hypertrophic CardioMyopathy
KEGG: Kyoto Encyclopedia of Genes and Genomes
MF: Molecular Function
ROC: Receiver Operating Characteristic
TP: True Positive.
