# Supplementary figures and images for: A comparative study of improvements Pre-filter methods bring on feature selection using microarray data
Source: Health Inf Sci Syst. 2014 Oct 16;2:7. doi: 10.1186/2047-2501-2-7 (PMC4340279; doi:10.1186/2047-2501-2-7)

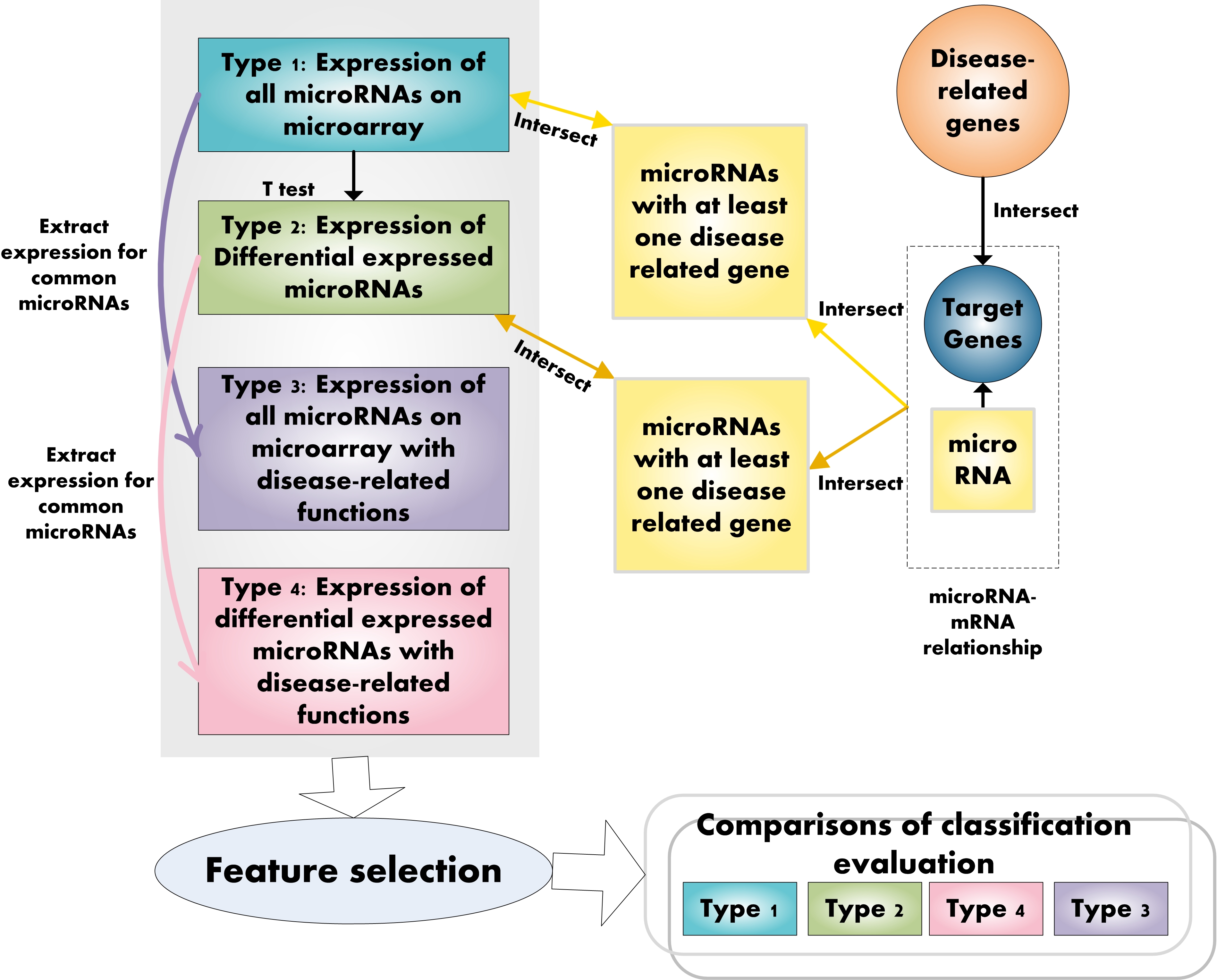

Supplement: Supplementary file 1 — Additional file 1: Framework of microRNA analysis in this study. (JPEG 2 MB) [file 13755_2014_17_MOESM1_ESM.jpeg]
